# Biochar-Enhanced Sulfur: Mechanistic Insights into a Novel and Effective Bactericide

**DOI:** 10.3390/nano15090697

**Published:** 2025-05-06

**Authors:** Yuanqi Peng, Lezhu Su, Meng Liu, Chen Zeng, Bo Xiang, Zhuoyao Xie, Zijing Hu, Nan Zhou

**Affiliations:** 1 Hunan Engineering Research Center for Biochar, Hunan Agricultural University, Changsha 410128, China; pengyuanqi@stu.hunau.edu.cn (Y.P.); sulezhu@stu.hunau.edu.cn (L.S.); liumeng0824@nwafu.edu.cn (M.L.); 1520002129@stu.hunau.edu.cn (C.Z.); 3187467939@stu.hunau.edu.cn (B.X.); xiezhuoyao@stu.hunau.edu.cn (Z.X.); huzijing@stu.hunau.edu.cn (Z.H.); 2 College of Chemistry and Materials Science, Hunan Agricultural University, Changsha 410128, China; 3 College of Resources, Hunan Agricultural University, Changsha 410128, China

**Keywords:** new pesticides, sulfur, biochar, antibacterial, redox reaction

## Abstract

The development of green, efficient, and stable pesticides for controlling agricultural pathogens remains a critical research focus. Elemental sulfur, although widely used for its bactericidal and insecticidal properties, suffers from aggregation, poor dispersibility, and limited contact with target organisms, restricting its effectiveness. In this study, we synthesized a novel biochar–sulfur composite by combining sustainable biochar with sulfur at low temperatures. The resulting material exhibited enhanced dispersibility and a five-fold increase in bactericidal efficacy compared to sulfur alone, as demonstrated in tests against *R. solanacearum* and *E. coli*. Additionally, the composite maintained 80% efficacy after five cycles of use, highlighting its favorable cyclic performance. Mechanistic studies revealed that biochar accelerates sulfur’s redox reaction, generating free radicals that drive efficient bactericidal action. This work provides a simple and sustainable approach for developing sulfur-based antimicrobial pesticides, offering new opportunities for sulfur utilization in agriculture.

## 1. Introduction

With the global population growth and the expansion of agriculture and animal husbandry, the threat posed by pathogenic bacteria in agricultural environments has intensified. These bacteria not only reduce crop yields and affect the quality of agricultural products but may also lead to public health issues [1,2,3]. In recent years, the expansion of vegetable cultivation areas has been accompanied by challenges, such as continuous cropping and contamination of irrigation water, which have increased the accumulation of pathogenic bacteria in the soil [4,5]. This has accelerated the development of bacterial crop diseases and made them difficult to control, thereby limiting the growth of the vegetable industry. Additionally, enteric pathogens in the soil, such as *E. coli*, may contaminate agricultural products and pose threats to human health [6,7]. Against the backdrop of worsening adverse climatic conditions, this biotic stress is expected to intensify [8,9], underscoring the importance of effective disease management to meet the projected increase in food demand by 2050 due to population growth [10].

Traditional approaches to managing agricultural pathogens often rely on chemical pesticides, such as copper-based compounds and antibiotics [11,12]. However, long-term use of these pesticides can lead to pesticide residues, environmental pollution, ecological imbalance, and threats to human health, while also promoting the development of resistance in pathogenic bacteria, thereby reducing their effectiveness [13]. Therefore, there is an urgent need to develop novel, efficient, and non-toxic green pesticides. Sulfur, which has been used for crop protection since ancient times and is considered safe [14], is not only one of the essential elements for plant growth [15], but also exhibits broad-spectrum antimicrobial potential against various plant pathogens [16,17]. Sulfur occupies an important position in agricultural chemistry, and research on its activity has primarily focused on developing sulfur-based formulations. When combined with systemic fungicides, sulfur can expand the range of antimicrobial activity and prolong its lifespan, demonstrating potential as an alternative fungicide in agriculture [18,19]. However, sulfur has several drawbacks, such as low water solubility, tendency to aggregate, low specific surface area for reactions, and susceptibility to loss, which limit its application [20]. Studies have shown that the antimicrobial activity of sulfur largely depends on particle size [21]. In recent years, efforts have been made to enhance the antimicrobial properties of sulfur by reducing its size to the nanoscale [22,23,24]. Various sulfur-based nanomaterials have been synthesized as potential antimicrobial agents, but most face challenges, such as complex synthesis processes, high costs, and limited antimicrobial activity [25,26].

Preparing sulfur-containing composite materials is one of the traditional methods to enhance the properties of elemental sulfur. The thermal diffusion method is commonly used to prepare carbon–sulfur composites, where sulfur is diffused into a carbon matrix at temperatures of 150–155 °C [27]. Biochar (BC), a carbonaceous material with abundant porosity, low cost, high stability, and environmental friendliness [28,29], has a high specific surface area that allows uniform dispersion of sulfur and enhances conductivity and specific surface area for reactions [30,31]. Its large pore volume effectively accommodates sulfur, preventing its detachment and improving stability, while its surface functional groups act as active sites to participate in or promote redox reactions [32]. Biochar derived from various biomass sources has shown excellent performance in lithium–sulfur battery applications [33], but its use in agricultural antibacterial research, particularly in carbon–sulfur composite materials, remains limited. The auxiliary role of biochar in enhancing the antibacterial properties of sulfur requires further investigation.

In this study, we innovatively selected the most common human foodborne pathogen, *Escherichia coli* (abbreviated as: *E. coli*), and the notorious plant pathogen *Ralstonia solanacearum* (abbreviated as: *R. solanacearum)*, which severely impacts solanaceous crop yields, as test subjects. We prepared a biochar material from chili pepper straw, an agricultural waste biomass and a green sustainable material. By low-temperature physical compounding of elemental sulfur with biochar, we innovatively fabricated a novel biochar–sulfur composite antibacterial material. We investigated its antibacterial properties against human and plant pathogens and analyzed redox reactions and radical generation within the material using various characterization techniques, thoroughly elucidating its bactericidal mechanism. To our knowledge, this is the first study to demonstrate that biochar–sulfur composites synthesized at low temperatures exhibit five-fold higher bactericidal efficacy than elemental sulfur alone, with exceptional recyclability (80% efficacy after 5 cycles). The mechanistic role of biochar in accelerating sulfur redox reactions to generate free radicals further distinguishes this work from prior reports. Results showed that physical compounding modification significantly enhanced the catalytic properties of elemental sulfur, and the material exhibited remarkable bactericidal activity and good recyclability. This work provides new theoretical insights and practical guidance for designing and developing sulfur-based antibacterial materials and lays a foundation for eco-friendly agricultural disease control and the broad application of biochar and inorganic materials in agriculture.

## 2. Materials and Methods

### 2.1. Chemicals and Reagents

Sublimated sulfur (S), sodium chloride (NaCl), Acetic Acid (CH_3_COOH), Potassium Iodate (KIO_3_), Potassium Iodide (KI), Sodium Thiosulfate Pentahydrate (Na_2_S_2_O_3_·5H_2_O), Ammonium Hydroxide (NH_4_OH), Anhydrous Ethanol (C_2_H_5_OH), Zinc Acetate (Zn(CH_3_COO)_2_), Glucose, Tryptone, Beef Extract and Yeast Extract are all analytically pure reagents provided by Aladdin Industrial Corporation, Riverside, CA, USA. Bacteria culture media were procured from Oubaoxing Biotech Co., Ltd, Beijing, China. Malondialdehyde assay kits were purchased from Shanghai Solebao Co., Ltd, Shanghai, China.

### 2.2. Preparation of the BC

In this study, we selected pepper straw as the biomass feedstock to fabricate materials designated as BC. Initially, the pepper straw was washed with water, dried in an oven at 60 °C for 48 h, cut into small pieces, and then pulverized followed by sieving through a 100-mesh screen. Under a nitrogen atmosphere, the biomass was heated in a tubular furnace (OTF-1200X, HF-KEJING Material Technology Co., Ltd., Hefei, China) to 800 °C (at a heating rate of 10 °C/min), held for 2 h, and the resulting carbon material was rinsed with distilled water and filtered until neutral to obtain BC.

### 2.3. Preparation of the BC@S and S

**Figure 1:**
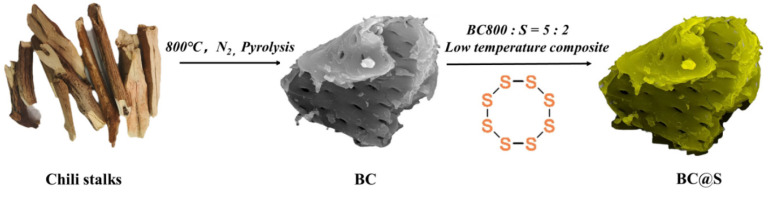
Schematic diagram of the preparation of BC and BC@S.

Subsequently, the biochar from the previous step was uniformly mixed with sulfur powder in a mortar at a ratio of 5:2, and the mixture was transferred into a weighing bottle, which was then sealed. The sulfur was first carbonized in a muffle furnace at 155 °C for 12 h to melt and infiltrate the biochar. Following this, the mixture was carbonized at 200 °C for 3 h to allow the sulfur within the biochar to sublime, yielding the BC@S composite material (Figure 1).

(1) $$\begin{array}{c} S\left(s\right)\to \;S\left(g\right) \end{array}$$

(2) $$\begin{array}{c} S\left(g\right)\to S_{surf}(s) \end{array}$$

### 2.4. Characterization

The surface morphology and elemental distribution of the materials were examined using a Scanning Electron Microscope (SEM, MIRA3 LMH, TESCAN, Brno, Czech Republic) and an Energy-Dispersive Spectrometer (EDS, Ultim Max 20, Oxford Instruments, Abingdon, UK). The particle size distribution in aqueous solutions was analyzed using a Laser Particle Size Analyzer (Mastersizer 3000, Malvern Panalytical, Worcestershire, UK). Surface elemental composition was determined using an X-ray Photoelectron Spectrometer (XPS, ESCALAB 250Xi, Thermo Fisher Scientific, Waltham, MA, USA). The crystal structure was investigated using an X-ray Diffractometer (XRD-6000, Shimadzu Corporation, Kyoto, Japan) with a 2θ scan range of 10 to 80 degrees. Functional groups in the range of 400 to 4000 cm^−1^ were determined using a Fourier Transform Infrared Spectrometer (FT-IR, Spectrum 65, PerkinElmer, Waltham, MA, USA). The specific surface area and pore size distribution of the materials were measured using the Specific Surface Area and Pore Size Analyzer (BET, SSA-6000, Biaode Electronics Technology Co., Ltd., Beijing, China). Free radicals were detected using an Electron Paramagnetic Resonance (EPR) spectrometer (EPR, JES-FA200, JEOL, Tokyo, Japan).

### 2.5. Evaluation of Antibacterial Effect

#### 2.5.1. Culture and Preparation of Bacteria

In this experiment, *R*. *solanacearum* (bacterial wilt pathogen) and *E. coli* were selected as the target bacterial strains. *E. coli* (batch number BNCC336902) and *R. solanacearum* (batch number BNCC335855) were both purchased from North China Bio-Union Biotechnology Co., Ltd, Beijing, China.

*R. solanacearum* was cultured in Nutrient Broth (NB) medium, while the *E. coli* strain was cultured in Luria–Bertani (LB) medium. Therefore, prior to preparing the bacterial suspensions, NB and LB liquid media and solid media were prepared separately. First, the bacterial strains were activated. In a disinfected laminar flow cabinet, an appropriate amount of the original bacterial culture (*E. coli* or *R. solanacearum*) was inoculated into conical flasks containing 40 mL of NB/LB liquid medium using an inoculating loop. The flasks were then incubated in a shaking incubator at constant temperatures of 37 °C (*E. coli*) and 30 °C (*R. solanacearum*) with a shaking speed of 150 rpm for 24 h (*E. coli*) and 48 h (*R. solanacearum*), respectively, to activate the bacterial cultures.

When the optical density (OD600) of the bacterial suspension exceeded 0.4 (±0.02), the suspension was centrifuged at 5000 rpm for 10 min using a benchtop high-speed centrifuge. The resulting pellet was washed with phosphate-buffered saline (PBS) to remove residual nutrients from the suspension. Subsequently, the bacteria were diluted in PBS to a concentration of 10^5^ cfu/mL, yielding the two bacterial suspensions required for the experiment. These suspensions were stored in a refrigerator for subsequent use.

To ensure bacterial viability, the integrity of the cell membrane was verified under a fluorescence microscope before each use of the bacterial suspension. Additionally, control groups were included in each experiment to confirm that the bacteria could be cultured successfully.

#### 2.5.2. Antibacterial Experiment

To evaluate the antibacterial efficacy of the prepared composite materials against *R. solanacearum* and *E. coli*, corresponding antibacterial experiments were conducted.

Antibacterial Experiment Procedure: In a 150 mL conical flask, 1 mL of bacterial suspension (10^5^ cfu/mL) and 49 mL of sterile distilled water were accurately measured (resulting in a final bacterial concentration of 2 × 10^3^ cfu/mL). Subsequently, identical amounts of different antibacterial materials were placed into 150 mL conical flasks, with a control group (no material added, only bacterial suspension and sterile distilled water). The flasks were sealed with parafilm and placed in a shaker (200 rpm). After reaching the predetermined antibacterial time, 200 μL of liquid from the conical flask was transferred to an appropriate amount of PBS for spread plate dilution and plating. The plated media were then incubated in a constant-temperature incubator until colonies formed (*E. coli*: 24 h, *R. solanacearum*: 48 h). The number of colonies on the plates was observed and counted, and the antibacterial efficiency was calculated using the control group as a reference. (The shaker and incubator were set to constant temperatures optimal for bacterial growth: *E. coli*: 37 °C, *R. solanacearum*: 30 °C.) Prior to the main experiments, preliminary experiments determined the dosage of antibacterial materials (1 mg/mL (0.05 g), 0.6 mg/mL (0.03 g), 0.2 mg/mL (0.01 g)) and the optimal antibacterial time (*E. coli*: 100 min, *R. solanacearum*: 160 min).

Reusability Experiment: this was carried out in a 150 mL conical flask by adding *E. coli* (10^5^ CFU/mL, 1 mL) to distilled water containing BC@S (1 mg/mL, 49 mL). The reaction was performed in a constant-temperature shaker (200 rpm, 37 °C). After reaching the reaction time, sampling and culturing were performed as described above. The antibacterial efficiency was calculated by comparing with the control group. This procedure was repeated nine times to evaluate the recyclable antibacterial performance of the material.

### 2.6. Statistical Analysis

To ensure the reliability and reproducibility of the experimental results, rigorous statistical analyses were conducted for all experiments. All experiments were performed with a minimum of three biological replicates (*n* ≥ 3), and the data were analyzed using Origin 2018 software. The specific statistical methods and results are detailed below.

#### 2.6.1. Antibacterial Efficiency Analysis

The antibacterial efficacy was assessed by the colony counting method, which involved spreading the treated bacterial suspension on agar plates, followed by incubation and enumeration of colony-forming units. To validate the significance of the experimental results, one-way analysis of variance (ANOVA) was employed to evaluate the overall differences among various treatment groups. All experimental data are presented as mean ± standard deviation (Mean ± SD). The formula for calculating antibacterial efficiency is as follows:

Antibacterial efficiency (%) = 1 − [(CFUcontrol − CFUtreatment)/CFUcontrol] × 100, where CFUcontrol and CFUtreatment are colony counts from untreated and treated groups, respectively.

#### 2.6.2. Determination of Sulfur Component Content in the Reaction System

The content of the sulfur component in the reaction system was determined using the indirect iodometric method, which is commonly employed for sulfur component detection. Detailed experimental procedures and calculation methods are provided in Supplementary Information S1.

## 3. Results and Discussion

### 3.1. Dispersion and Surface Morphology Analysis

**Figure 2:**
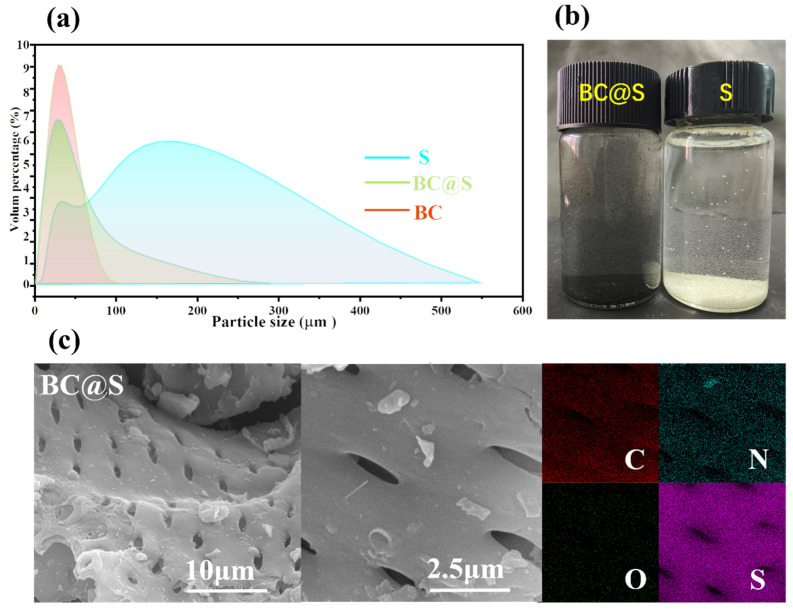
Particle size distributions of BC@S, BC and S measured with aqueous liquid as a medium (**a**), Macroscopic states of BC@S and S particles in the aqueous liquid (**b**), SEM images and EDS analysis of BC@S (**c**).

The dispersibility of materials in water is fundamental for their contact with microorganisms and their subsequent bactericidal action, which is conducive to enhancing the utilization rate of the materials. Initially, the auxiliary role of biochar in improving the dispersibility of materials in aqueous media was explored. A laser particle size analyzer was employed to test the dispersion states of BC, S, and the BC@S composite material in water, as shown in Figure 2a. The dispersibility of BC@S and BC in water was significantly better than that of S, with the majority of particle sizes distributed below 50 μm. Observations from Figure 2b also clearly reveal that a large number of fine particles of BC@S are uniformly dispersed in the aqueous medium. In contrast, most of the particle sizes of S are distributed above 150 μm, likely due to the tendency of S to form aggregates in water, with some S particles encapsulated within these aggregates. It can also be directly observed that severe aggregation occurs in the aqueous medium, with most particles aggregating into larger clusters that settle at the bottom of the beaker, losing the opportunity to fully contact *E. coli* in the water, which may affect their bactericidal effect. In comparison, when BC@S particles are added to the test medium, their good dispersibility in water allows for the exposure of more surface area to contact *E. coli* in the water, which may contribute to enhancing their bactericidal capability.

To visually observe how biochar and sulfur are combined in the BC@S composite material and to explain the reason for the improved dispersibility of the BC@S material due to the presence of biochar, the morphologies of BC and BC@S composite materials were investigated using a scanning electron microscope (SEM). As shown in Figure S1, before sulfur loading, the surface of the BC material is smooth with a large number of round pores, which may be conducive to accommodating molten sulfur. After grinding and sulfur loading by melting, the microstructure of the BC@S composite material is shown in Figure 2c, which is similar to the morphology of BC, with almost no visible sulfur particles on the surface. The energy-dispersive spectroscopy (EDS) image (Figure 2c) shows that sulfur is densely and uniformly distributed on the surface, which can be interpreted as uniform loading of sulfur on the material surface, possibly due to the biochar acting as a carrier, enhancing the dispersibility of sulfur [33]. To ascertain the sulfur content in the BC@S composite material, elemental analysis (EA) was conducted on BC@S and BC, as shown in Table 1. The sulfur content of BC is only 0.57% and, after sulfur loading, the sulfur content reached 27.39%, further indicating the successful preparation of a carbon–sulfur composite with a high sulfur content. To elucidate the auxiliary role of biochar in enhancing the dispersibility of BC@S, elemental analysis (EA) was performed on the materials. Generally, the O/C and (O + N)/C molar ratios in elemental analysis are indicators of the polarity and chemical reactivity of carbon materials [34,35]. The O/C, (O + N)/C ratios for BC and BC@S are 0.35, 0.37 and 0.24, 0.26, respectively. After sulfur loading, the ratios for BC@S have decreased relative to BC but still remain relatively high. This indicates that the polarity of the composite material is significantly enhanced after the introduction of biochar. This allows the carbon material surface to form stronger interactions with polar molecules or substances. Carbon materials with a high O/C ratio are more easily dispersed uniformly in polar solvents, such as water [34], forming a stable dispersion system, which is consistent with the results from the laser particle size analyzer. Additionally, the nitrogen content of both materials reached 1.17%, suggesting that the material surface may contain a significant number of nitrogen-containing functional groups. Nitrogen and oxygen-containing functional groups can act as active sites [36], promoting electron transfer and enhancing reactivity to improve the bactericidal performance of the material.

### 3.2. Investigation of the Bactericidal Performance of Sulfur Enhanced by Biochar

**Figure 3:**
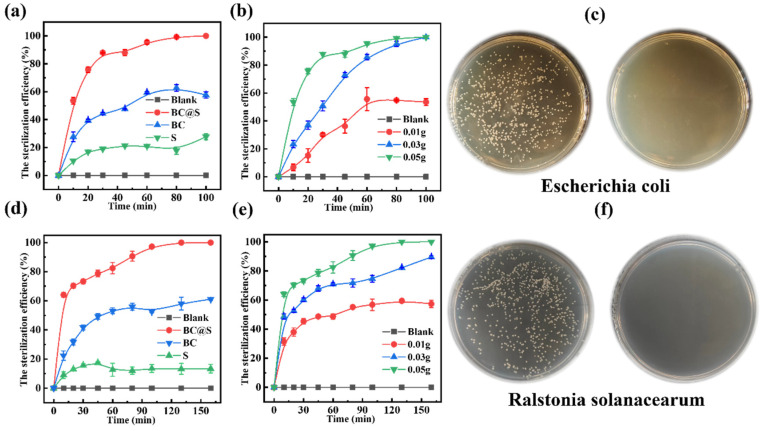
Comparison of bactericidal efficiency among different materials and bacterial strains. Bactericidal efficiency of S (elemental sulfur), BC (Biochar), BC@S (Biochar–Sulfur composite material), and the blank (control group) against *E. coli* (**a**), *R. solanacearum* (**d**) at an addition amount of 0.05 g. Bactericidal efficiency of BC@S at different addition amounts (0.05 g, 0.03 g, and 0.01 g) against *E. coli* (**b**), *R. solanacearum* (**e**), Comparison of bacterial colony counts on agar plates between the control group (left) and the treatment group (BC@S, 0.05 g, right) for *E. coli* (**c**), *R. solanacearum* (**f**).

An exploration took place of the bactericidal performance of sulfur augmented by biochar. Initially, the bactericidal effects of BC (biochar), S (elemental sulfur), and BC@S (biochar–sulfur composite material) on *E. coli* were investigated. The results, as depicted in Figure 3a, demonstrated that, after a 100-minute experimental period, the bactericidal efficacy of the carbon–sulfur composite BC@S was significantly superior to that of BC and S, with the order of effectiveness being BC@S (100.00%) > BC (57.68%) > S (27.77%). Compared to S, the bactericidal effect of the composite material was enhanced by a factor of five. It is hypothesized that the composite material’s improved dispersibility allows for better contact with bacteria. Additionally, the biochar’s efficient electron transfer properties facilitate redox reactions involving sulfur, significantly enhancing the material’s bactericidal capability and achieving efficient killing. In contrast, the strong hydrophobicity of S and its severe aggregation significantly reduce the effective surface area for contact with bacteria and subsequent reaction, resulting in the weakest bactericidal effect. This outcome is consistent with the inferences drawn from laser particle size analysis. To further elucidate the impact of BC@S dosage on the removal rate, bactericidal experiments with varying amounts of carbon (0.05 g (1 mg/mL), 0.03 g (0.6 mg/mL), 0.01 g (0.2 mg/mL)) were conducted. As shown in Figure 3b, a dosage of 0.05 g achieved a 100.00% bactericidal rate after 80 min. When the dosage was 0.03 g, complete bactericidal action was also achieved within 100 min. However, with a dosage of 0.01 g, the bactericidal process was not completed, reaching only a 53.56% bactericidal rate after 100 min. This indicates a positive correlation between bactericidal efficiency and the amount of carbon material added. To visually assess the bactericidal effect of BC@S, agar plate colony counting was used to evaluate *E. coli*, as shown in Figure 3c. The left side represents the untreated control group, while the right side displays the effect after 100 min of BC@S treatment. It can be observed that, in the absence of BC@S, *E. coli* grew well, indicating a strong survival and living capability for this type of bacteria. After BC@S treatment, no colonies appeared on the plate, clearly demonstrating the composite material’s potent bactericidal effect. Subsequently, the same bactericidal experiments were conducted on *R. solanacearum*. As shown in Figure 3d, after a 160-minute experiment, the bactericidal intensities of the three materials against *R. solanacearum* were consistent with the expected results, with the order being BC@S (100.00%) > BC (61.09%) > S (13.38%). BC@S still exhibited the best bactericidal ability, achieving a 100% bactericidal effect, while S showed the poorest effect, consistent with the results against *E. coli*.

Similarly, different doses of BC@S (0.05 g (1 mg/mL), 0.03 g (0.6 mg/mL), 0.01 g (0.2 mg/mL)) were tested, and the results are shown in Figure 3e, where the bactericidal efficiency was positively correlated with the amount added. It can be observed that, when the dosage was 0.05 g, the bactericidal efficiency of BC@S reached 100.00%, when the dosage was 0.03 g, it reached 89.56% and, when the dosage was 0.01 g, its bactericidal effect was only 57.41%. After treatment with BC@S for 160 min, the spread plate (Figure 3f) showed no colonies of *R. solanacearum*. Compared with the control group, the composite material still demonstrated a high bactericidal efficiency. The aforementioned bactericidal results indicate that the BC@S composite material has strong bactericidal activity, and the presence of biochar assists in enhancing the bactericidal activity of sulfur, showing potential for efficiently combating plant pathogenic bacteria.

To verify the stability of BC@S, a sterilization cycle experiment with *R. solanacearum* was conducted using 0.05 g (1 mg/mL) of the composite material. After each sterilization cycle, samples were taken, spread on agar plates, and an equal amount of bacterial suspension was added to continue the sterilization process. A total of 9 cycle experiments were performed (Figure S2). It can be observed that, during the first four-cycle experiments, the bactericidal rate of the composite material remained at 100%, indicating that the material has excellent stability and strong bactericidal ability. When the fifth cycle was reached, the bactericidal effect of the material decreased to 80%. Observation of the reaction system after sterilization revealed that a small part of the material had aggregated and formed flocs. Continuing the experiment, the bactericidal effect of the material continued to decline. During the eighth cycle, the bactericidal effect was only 12%, and, by the ninth cycle, the material showed no bactericidal effect. Observation of the sterilization system revealed that most of the material had formed larger flocs (Figure S3). It is speculated that the reason for the decreased bactericidal efficiency may be the aggregation of inactive bacteria and material, which reduced the contact area between the material and bacteria, thereby limiting the bactericidal effect.

### 3.3. Investigation into the Mechanism of Bactericidal Action Augmented by Biochar-Supported Sulfur

#### 3.3.1. Changes in the Surface Properties of BC@S

**Figure 4:**
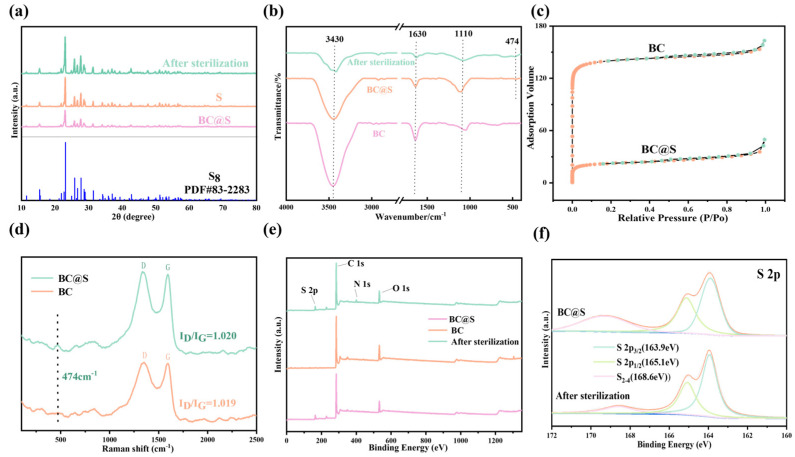
(**a**) XRD patterns of BC@S, S and after sterilization, (**b**) FT–IR patterns of BC@S, BC and after sterilization, (**c**) BET patterns of BC@S, BC, (**d**) Roman patterns of BC@S, BC, (**e**) XPS survey spectrum of BC@S, BC and after sterilization, (**f**) S 2p of XPS of BC@S and after sterilization.

XRD analysis (Figure 4a) indicated that the surface of the BC@S sample prior to sterilization primarily consisted of S_8_ crystals (JCPDS No. 83-2283), with no other extraneous peaks present, further confirming the existence of sulfur within the material. The XRD spectrum of the material after sterilization (Figure 4a) showed no significant changes on the surface, suggesting that the reaction may not occur directly on the material’s surface. Fourier-transform–infrared spectroscopy (FT–IR) further validated this hypothesis, as shown in Figure 4b. Both BC and BC@S exhibited a prominent absorption peak near 3430 cm^−1^, primarily attributed to the stretching vibrations of -OH groups present in alcohols and phenols, either free or in intermolecular associations [37]. Additionally, three absorption peaks observed at wavelengths of 1630 cm^−1^, 1383 cm^−1^, and 1110 cm^−1^ correspond to the stretching vibrations of C=O, -RCOO, and C-O, respectively, indicating that the BC@S surface contains a significant number of oxygen-containing functional groups, which enhances its polarity and facilitates good dispersion in aqueous media, consistent with the results from laser particle size analysis [38]. Furthermore, the peak near 474 cm^−1^, resulting from the vibration of S–S bonds [39], confirms the successful loading of sulfur on the material surface, aligning with the XRD characterization results. Notably, the aforementioned oxygen- and sulfur-containing functional groups remained present after sterilization without any significant changes, further supporting the inference that the reaction may not have occurred directly on the material surface. BET analysis was conducted to verify the porosity changes of the material before and after sulfur loading. As shown in Figure 4c, both BC and BC@S exhibit type IV adsorption isotherms, indicating a mesoporous structure. BC demonstrates a higher specific surface area, while BC@S has a lower specific surface area, likely due to pore filling by elemental sulfur. Nevertheless, the pore structure of BC@S is more uniform, which enhances sulfur dispersion and boosts antibacterial performance. Raman spectroscopy reveals that both BC and BC@S exhibit distinct D bands (~1350 cm^−1^) and G bands (~1590 cm^−1^). As shown in Figure 4d, the I_D_/I_G_ values are 1.019 for BC and 1.020 for BC@S, indicating significant disorder and a high density of defects and active edge sites, which may participate in catalytic reactions. Additionally, BC@S shows a sulfur characteristic peak at 474 cm^−1^, confirming the presence of elemental sulfur. These findings align with XRD and BET results, further confirming successful sulfur incorporation. In summary, it is confirmed that sulfur exists within the composite material, dispersed on the surface of the carbonaceous material, and it is speculated that sulfur may primarily be in a physically loaded state in conjunction with biochar, suggesting that the reactions during the sterilization process may not have directly taken place on the surface of the material.

In order to explore the changes in the functional groups of BC@S materials before and after sterilization, X-ray photoelectron spectroscopy (XPS) analysis was performed. The results are depicted in Figure 4e, where it is evident that the material surface exhibits four diffraction peaks for C 1s (284.4 eV), N 1s (399.4 eV), O 1s (531.4 eV), and S 2p (164.2 eV). The high-resolution spectrum of C 1s is shown in Figure S4, with the binding energy at 284.7 eV corresponding to C-C. In addition, there are three smaller component peaks at 285.3 eV, 286.5 eV and 289.3 eV, corresponding to C=C, C-O and O-C=O, respectively, which may be derived from oxygen-containing functional groups on the surface of the carbon material. After sterilization, the proportions of C-C and C=C bonds decreased, while the proportions of C-O and O-C=O bonds increased. This suggests that the carbon framework underwent oxidation, likely due to reactions with reactive oxygen species (ROS) such as ^•^OH and O_2_^•−^ generated during sterilization [40]. These changes enhance the material’s polarity and reactivity. The high-resolution spectrum of O 1s is shown in Figure S4, with the binding energy at 531.5 eV,532.4 eV and 533.5 eV, corresponding to S=O, C-O and S-O. A decrease in S=O and C-O bonds alongside an increase in S-O bonds was observed post-sterilization. This indicates that sulfur species were further oxidized, contributing to the material’s oxidative capacity and potentially increasing its interaction with microbial cells [41]. Furthermore, the high-resolution N 1s spectrum (Figure S4) of both materials can be well resolved into five peaks with binding energies of 398.3, 399.8, 400.7, 401.7, and 403.3 eV, corresponding to pyridinic-N, amino-N, pyrrolic-N, graphitic-N, and oxidized-N, respectively (Figure S4). Nitrogen-doped carbon materials possess excellent catalytic properties; therefore, the significant active role of nitrogen-containing groups in redox reactions cannot be overlooked [42,43]. Pyridinic, amino, and graphitic nitrogen proportions decreased after sterilization, while pyrrolic and oxidized nitrogen proportions increased. This shift implies that nitrogen functional groups participated in redox reactions, possibly facilitating electron transfer and enhancing catalytic activity [44]. It is speculated that the synergistic effect between sulfur and nitrogen-containing groups on biochar likely contributes to the redox performance of the material [45]. The high-resolution S 2p spectrum is shown in Figure 4f. The peaks at 163.9 eV, 165.1 eV, and 168.6 eV are attributed to the S 2p2/3, S 2p1/2, and S_2-4_ components [33], respectively, confirming that sulfur is dispersed within the pores of BC to form a composite material. The morphology of sulfur on the material surface did not change after sterilization, indicating that the composite material has excellent stability. However, the content of short-chain S_2-4_ components decreased significantly, from 26.92% before sterilization to 14.46%, suggesting some loss of sulfur from the material surface. This leads to the speculation that the sterilization mechanism may involve sulfur on the composite material surface undergoing redox reactions with the assistance of biochar, completing the sterilization, and there may be sulfur components present in the sterilization system.

#### 3.3.2. Changes in Sulfur Composition in the System

To explore the changes in sulfur within the system before and after sterilization, the sulfur components in the system before and after sterilization were detected using the indirect iodometry method. First, BC@S was added to the system without bacteria and reacted for two hours, then samples were taken and filtered for detection. The same method was applied in the system with bacteria present, reacting for two hours and taking samples. The test results are shown in Table 2, where it can be seen that, before and after sterilization, three main sulfur-containing components, SO_4_^2−^, S^2−^, and sulfur oxides (S_x_O_y_), were detected in the system. The results indicate that, in the absence of bacteria, the concentration of SO_4_^2−^ was 0.1392 g·L^−1^, S^2−^ was 0.0483 g·L^−1^, and S_x_O_y_ reached 0.3608 g·L^−1^. After the addition of bacteria and a three-hour reaction, the concentrations of SO_4_^2−^ and S_x_O_y_ both increased. It is speculated that the possible reaction process is that, after BC@S is introduced into the aqueous system, the sulfur on the surface of the material undergoes redox reactions in the aqueous system with the catalytic action of biochar, resulting in highly dispersed sulfur reacting with water to produce a large amount of sulfur oxides and sulfate ions, and releasing electrons.

**Figure 5:**
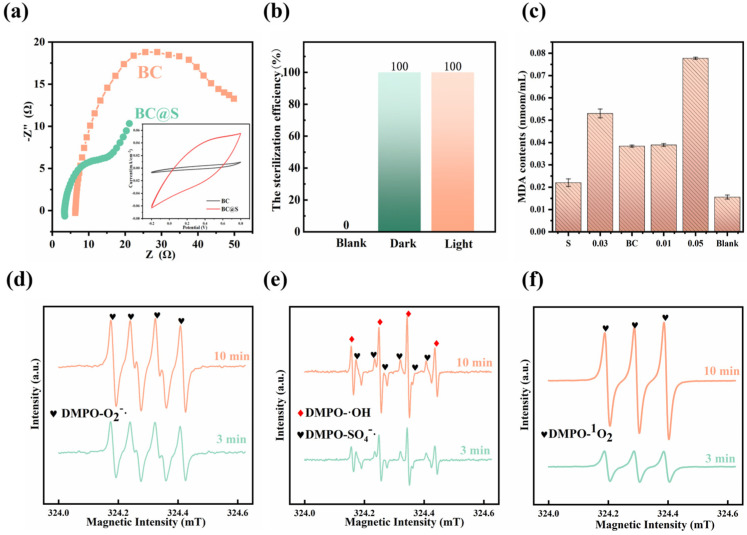
(**a**) Electrochemical performance test of BC and BC@S, (**b**) Comparison of sterilization under light/dark conditions, (**c**) MDA content change of different materials after sterilization, (**d**–**f**) The electron paramagnetic resonance (EPR) spectrum in BC@S.

The aforementioned research indicates that biochar does not directly participate in the reaction within the composite material but rather serves a more catalytic role. To investigate the redox properties of the material and explain the promotional effect of biochar on the reaction of sulfur, electrochemical tests were conducted on BC and BC@S composite materials. Cyclic voltammetry (CV curves) was used to evaluate the specific capacitance and other performance characteristics of BC@S and BC at a scan rate of 0.1 V·S^−1^. The results, as shown in Figure 5a, demonstrate that BC@S has a larger specific capacitance and current density in the electrolyte solution, and it exhibits stronger reduction (minimum value) and oxidation potentials (maximum value), confirming its redox capability [46]. Electrochemical impedance spectroscopy (EIS) was employed to study the electrochemical kinetics of BC@S and BC, with each material’s EIS consisting of high-frequency and low-frequency regions. The X-intercept in the high-frequency region of the material is related to the material’s inherent Ohmic resistance. It can be observed that BC@S has a lower charge transfer resistance, and the diffusion line in the low-frequency region indicates that ion diffusion on the surface of BC@S is rapid, with a high electron transfer rate [47]. Thus, due to the catalytic assistance of biochar, BC@S possesses superior electrochemical performance, providing the possibility for catalyzing the generation of free radicals from O_2_ and H_2_O.

#### 3.3.3. Changes in Bacteria and Bactericidal Mechanism

Considering the material’s superior redox and catalytic properties, we aimed to verify whether bacterial death was caused by redox reactions and free radical production. To this end, we selected E. coli as the target bacterium and examined the malondialdehyde (MDA) content in E. coli after sterilization with different materials. This approach allowed us to explore the potential mechanisms of bacterial death. It is well-established that microorganisms experience lipid peroxidation as a consequence of cellular stress, which can lead to structural damage and death. MDA represents the ultimate byproduct of lipid peroxidation within cellular membranes, serving as a biomarker for the extent of lipid peroxidation and, by extension, providing an indirect measure of cellular damage [48]. Figure 5c illustrates the levels of MDA in *E. coli* both prior to and subsequent to exposure to various sterilizing agents. The comparative analysis reveals a notable elevation in MDA levels post-treatment with the sterilizing agents, with the sequence of damage being BC@S surpassing S and BC. This pattern suggests that BC@S exerts a more pronounced detrimental effect on *E. coli* compared to S and BC. The observed increase in MDA content is indicative of the oxidative stress induced by the sterilizing materials, which is likely attributable to the generation of reactive oxygen species (ROS) and the ensuing redox reactions that lead to the formation of peroxides and cellular structural disruption. To verify whether the composite material can produce free radicals for sterilization, an EPR test was conducted (Figure 5d–f). The EPR spectrum using DMPO as a spin-trapping agent revealed peaks for ^1^O_2_, ^•^OH, SO_4_^−•^, and O_2_^•−^ after the addition of BC@S composite catalytic material to the aqueous system for 3 min. This indicates that BC@S can not only produce SO_4_^−•^ through its own redox reactions but can also catalyze the generation of free radicals and singlet oxygen from O_2_/H_2_O during the reaction, demonstrating that the material can indeed sterilize by producing free radicals. To rule out the influence of light on the sterilizing effect of the material, sterilization experiments were conducted under both illuminated and dark conditions(Figure 5b). Under otherwise identical conditions, equal amounts of the composite material were used for sterilization in both light and dark conditions. The comparison revealed that the sterilization efficiency was 100% in both conditions, thus, light is not a factor affecting sterilization.

Based on the aforementioned experimental results and analysis, the bactericidal mechanism of the composite material has been deduced. The possible reaction process is as follows: the antibacterial mechanism of the BC@S composite material is attributed to several key factors. Initially, redox reactions occur when the sulfur in BC@S interacts with biochar in an aqueous solution, generating highly oxidative free radicals (such as ^•^OH, SO_4_^−•^, and O_2_^•−^). Secondly, the surface of BC@S is rich in oxygen-containing functional groups (e.g., -OH, C=O, C-O) and nitrogen-containing functional groups (e.g., pyridinic N, amino N, pyrrolic N, graphitic N, and oxidized N). These groups enhance the material’s polarity and catalytic properties, further promoting radical formation. Additionally, the porous structure and high dispersibility of BC@S increase its contact area with bacteria, significantly improving antibacterial efficiency. Experimental results indicate that BC@S exhibits excellent antibacterial performance against *E. coli* and *R. solanacearum*, with both the MIC and MBC values determined to be 0.6 mg/mL and 1 mg/mL, respectively. In summary, the BC@S composite material achieves highly efficient bacterial inactivation through a combination of mechanisms: the generation of highly reactive free radicals via redox reactions, the polarity and catalytic activity of surface functional groups, the high dispersibility of its porous structure, and the synergistic effects between biochar and sulfur. The specific reaction equations are as follows:

(3) $$\begin{array}{c} \;{BC}_{surf}-S+O_{2}+H_{2}O\;\to S_{x}O_{y}\;+e^{-} \end{array}$$

(4) $$\begin{array}{c} {BC}_{surf}-S+e^{-}\to S^{2-} \end{array}$$

(5) $$\begin{array}{c} \;S^{2-}+O_{2}\to S_{x}O_{y}\;+e^{-} \end{array}$$

(6) $$\begin{array}{c} \;S_{x}O_{y}\;+O_{2}\to {SO}_{4}^{2-}+{SO}_{4}^{∙-}+e^{-} \end{array}$$

(7) $$\begin{array}{c} O_{2}+e^{-}\to \;{∙O}_{2}^{-} \end{array}$$

(8) ∙O2−+2 H2O → H2O2+2 OH−+O21

(9) $$\begin{array}{c} H_{2}O_{2}+e^{-}\to \;∙OH+{OH}^{-} \end{array}$$

## 4. Conclusions

In summary, a novel sulfur-based antibacterial material for the control of harmful bacteria has been successfully developed through a low-temperature composite method using chili stalk biochar and sulfur. Characterization by laser particle size analyzer, XRD, SEM, XPS, and electrochemical techniques confirmed that the composite material primarily consists of sulfur and a biochar matrix, which are mainly combined physically. The auxiliary role of the biochar endows the composite material with good dispersibility and higher reactivity. Bactericidal performance tests against *R. solanacearum* and *E. coli* demonstrated that the material can complete sterilization within 160 min, exhibiting high stability and sustained usability. After five cycles, the sterilization performance remained at 80%, confirming that, with the assistance of chili pepper straw biochar, the bactericidal efficacy of sulfur was enhanced by five times. Subsequent investigation into the bactericidal mechanism revealed that the biochar in the composite material can aid in intensifying the redox reactions involving sulfur, generating active free radicals, achieving efficient sterilization.

## Figures and Tables

**Table 1:** Element content of BC and BC@S.

	Elemental Composition (%)					Atomic Ratio (%)		
	N	C	H	O	S	(O + N)/C	H/C	O/C
BC	1.17	70.81	2.43	25.02	0.57	0.37	0.03	0.35
BC@S	1.18	56.42	1.30	13.72	27.39	0.26	0.02	0.24

**Table 2:** Changes in sulfur content.

	S^2−^	S_x_O_y_	SO_4_^2−^
Before (g·L^−1^)	0.0483 ± 0.0032	0.3608 ± 0.0173	0.1392 ± 0.0355
After (g·L^−1^)	0.0464 ± 0.0016	0.3762 ± 0.0151	0.1444 ± 0.0216

## Data Availability

Data are contained within the article and Supplementary Materials.

## Supplementary Materials

The following supporting information can be downloaded at: https://www.mdpi.com/article/10.3390/nano15090697/s1, Figure S1: SEM patterns of BC; Figure S2: Sterilization cycle test of BC@S; Figure S3: BC@S in the system after nine sterilization cycles; Figure S4: XPS spectrum of C 1s, O 1s and N 1s spectrum of BC@S (**a**–**c**) and After sterilization (**d**–**f**).
