# Supplementary material for: Biochar-Enhanced Sulfur: Mechanistic Insights into a Novel and Effective Bactericide
Source: Nanomaterials (Basel). 2025 May 6;15(9):697. doi: 10.3390/nano15090697 (PMC12073505; doi:10.3390/nano15090697)
Supplement: Supplementary file 1 [file nanomaterials-15-00697-s001.zip › nanomaterials-3512738-supplementary.pdf]

Supplementary Information

Biochar-Enhanced Sulfur: Mechanistic Insights into a Novel and Effective Bactericide

Yuanqi Peng[1,2], Lezhu Su[1,2], Meng Liu[1,2], Chen Zeng[1,2], Bo Xiang[1,2], Zhuoyao Xie[1,2], Zijing Hu[1,2], Nan Zhou[1,2,*]

[1] Hunan Engineering Research Center for Biochar, Hunan Agricultural University, Changsha 410128, China

[2] College of Chemistry and Materials Science, College of Resources, Hunan Agricultural University, Changsha 410128, China

[*] Corresponding author: Prof. Nan Zhou, Email: zhounan@hunau.edu.cn;

S1 Materials and methods

1. The experimental method for the determination of sulfur component content by indirect iodometry.

1.1 Reagents.

Zinc acetate solution: 50 g/L, weigh 50 g of analytical pure zinc acetate into a 500 mL beaker, dissolve in water and transfer to a 1L reagent bottle, add 5.0 mL of glacial acetic acid to inhibit hydrolysis; Starch indicator: 5.0 g/L, weigh 0.5 g of starch into a 250 mL beaker, mix with 10.0 mL of cold water, stir and add 90 mL of boiled water, continue boiling for 5 minutes, after cooling, store in a 100 mL reagent bottle for later use; Formaldehyde solution: with a mass fraction of 36%; Ammonia water: with a mass fraction of 28%; $Na_2S\cdot 9H_2O$ standard solution: 0.0100 mol/L, weigh 2.4018 g of $Na_2S\cdot 9H_2O$, dilute and dissolve to make up volume in a 1L brown volumetric flask, standardized with iodine standard solution; $Na_2S_2O_3$ standard solution: 0.0100 mol/L, weigh 1.2604 g of anhydrous $Na_2S_2O_3$, dilute and dissolve to make up volume in a 1L brown volumetric flask, standardized with iodine standard solution; Glacial acetic acid, anhydrous ethanol, potassium iodide, sodium hydroxide, aluminum hydroxide, KIO_3, and $Na_2S_2O_3$ are standard reagents, all other reagents are of analytical purity, the experimental water is deionized water, aluminum hydroxide and aluminum sulfate solutions are obtained from Aladdin Industrial Company.

1.2 Preparation and Calibration of Standard Solutions.

Iodine standard solution: 0.00100 mol/L, weigh 0.07134 g of standard KIO_3 dried at 105 °C and 10 g of analytical pure KI, dissolve in water and dilute to volume in a

1L brown volumetric flask, mix well; this solution has a KIO_3 concentration of 0.00033 mol/L, equivalent to an iodine standard solution with a $c(I_2)$ of 0.00100 mol/L. It should be noted that under neutral conditions, KIO_3 and KI can stably exist without undergoing chemical reactions. Therefore, before the test begins, as acetic acid has not been added, the iodine element in the iodine standard solution still exists in the form of KIO_3 and KI, without the generation of elemental iodine, which can avoid the slight changes in iodine content caused by the unstable nature of elemental iodine. After the test begins, with the addition of acetic acid solution, the standard KIO_3 and KI can quantitatively generate elemental iodine and participate in the reaction under acidic conditions.

$Na_2S_2O_3$ standard solution: 0.0100 mol/L, weigh 2.32 g of analytical pure sodium sulfite decahydrate ($Na_2S_2O_3 \cdot 5H_2O$) and dissolve in water that has been boiled and cooled, then dilute to volume in a 1L brown volumetric flask and mix well.

Calibration of $Na_2S_2O_3$ standard solution: Take 15.0 mL of glacial acetic acid and add it to an iodine flask containing 100 mL of water, then pipette 20.00 mL of iodine standard solution and titrate with $Na_2S_2O_3$ standard solution until the solution turns light yellow, add 2.0 mL of starch indicator, and continue titrating until the blue color just disappears. The titration volume is V(mL), then the concentration of $Na_2S_2O_3$, $c(Na_2S_2O_3)$, is 0.0100 mol/L.

1.3 Determination of Sulfur Components.

Using a pipette, transfer 10.00 mL of the filtered solution from the system before and after the reaction into separate 100 mL volumetric flasks and make up to volume with water. Take 25.00 mL of this diluted solution and place it into a 500 mL iodine

flask that already contains 100 mL of water, 20.00 mL of iodine standard solution, and 15.0 mL of glacial acetic acid. Cover the flask, place it in a dark place for about 3 minutes, then titrate the excess iodine with $Na_2S_2O_3$ standard solution. When the solution turns a light yellow, add 20 mL of starch indicator and continue titrating until the blue color fades. Add drops quickly while shaking the flask until the blue color disappears, marking the endpoint. The volume of $Na_2S_2O_3$ standard solution used for the titration is V_1(mL).

Transfer 20.00 mL of the primary dilution into a 100 mL volumetric flask, add 10.0 mL of anhydrous ethanol to slow down the oxidation of S_xO_y (Defined as sulfur oxides, including $S_2O_3^{2-}$ and SO_3^{2-}) by oxygen in the air, shake well, then add 5.0 mL of zinc acetate solution to precipitate SO_4^{2-}, shake well, add 5.0 mL of ammonia water to dissolve the $Zn(OH)_2$ precipitate, shake well again, and make up to the mark with water. Filter the precipitate using a double-layer slow filtration paper and a dry funnel, and take 25.00 mL of the filtrate (equivalent to 1.00 mL of the original solution) and add it to a 500 mL iodine flask containing 100 mL of water, 2000 μL of iodine standard solution, and 15.0 mL of glacial acetic acid, shaking while adding. Cover the flask and let it stand in the dark for about 3 minutes. Titrate the excess iodine with $Na_2S_2O_3$ standard solution. When the solution turns a light yellow, add 2.0 mL of starch indicator and continue titrating until the blue color fades. Shake the flask quickly and add the solution drop by drop until the blue color disappears, marking the endpoint. The volume of $Na_2S_2O_3$ standard solution used for the titration is V_2(mL).

1.4. Calculation of Results.

The content of the three sulfur components in the test solution can be calculated based on the volume of $Na_2S_2O_3$ standard solution consumed in the two-step titration. The results are expressed in units of $g{\cdot}L^{-1}$. The calculation formula is as follows:

$$c(S^{2-}) = c(Na_2S_2O_3) * (V_2-V_1) * 32/2 \quad (1)$$
$$c(S_2O_3^{2-}) = [2 * c(I_2) * 20 - c(Na_2S_2O_3) * (V_2-V_1)] * 112 \quad (2)$$
$$c(SO_3^{2-}) = c(Na_2S_2O_3) * (V_2-V_1) * 80/2 \quad (3)$$
$$c(SO_4^{2-}) = (V_2-V_1) * c(Na_2S_2O_3) * 96/2 \quad (4)$$

In the formula: : *$c(S^{2-})$,$c(S_2O_3^{2-})$,$c(SO_3^{2-})$,$c(SO_4^{2-})$ represent the mass concentrations of* S^{2-}、$S_2O_3^{2-}$、SO_3^{2--}、SO_4^{2-} in the sample, respectively, in units of g/L. V_1 and V_2 are the volumes of the standard $Na_2S_2O_3$ solution consumed in the two titration steps, in units of mL.

32 is the molar mass of S^{2-}, in g/mol.

112 is the molar mass of $S_2O_3^{2-}$, in g/mol.

80 is the molar mass of SO_3^{2-}, in g/mol.

96 is the molar mass of SO_4^{2-}, in g/mol.

All experiments were triplicated. Data are presented as mean ± SD.

S2 Results and discussion

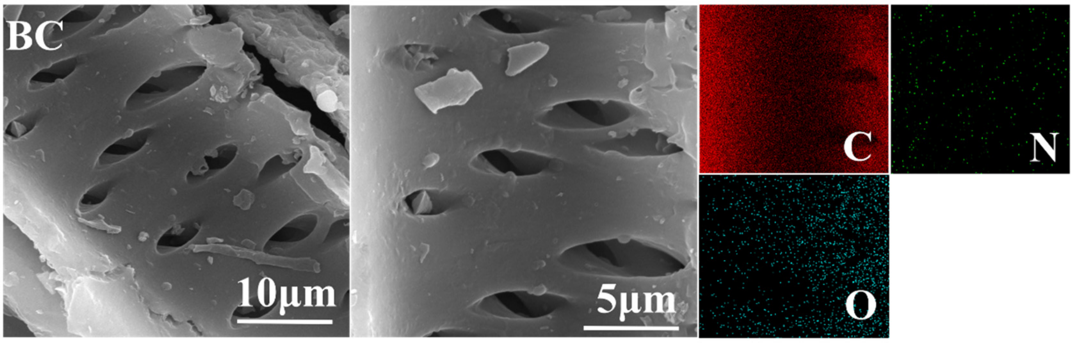

Figure S1. SEM patterns of BC.

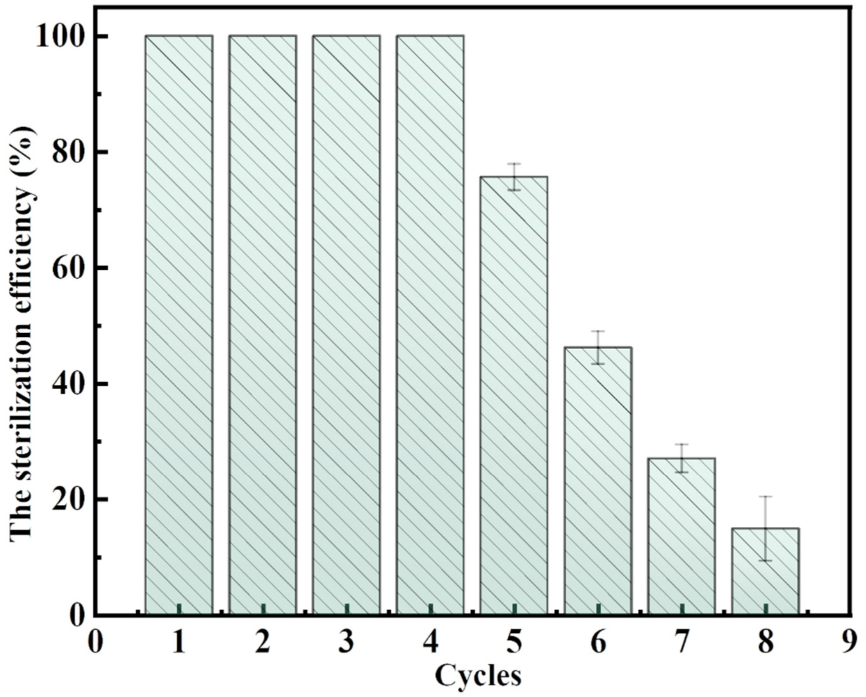

Figure S2. Sterilization cycle test of BC@S.

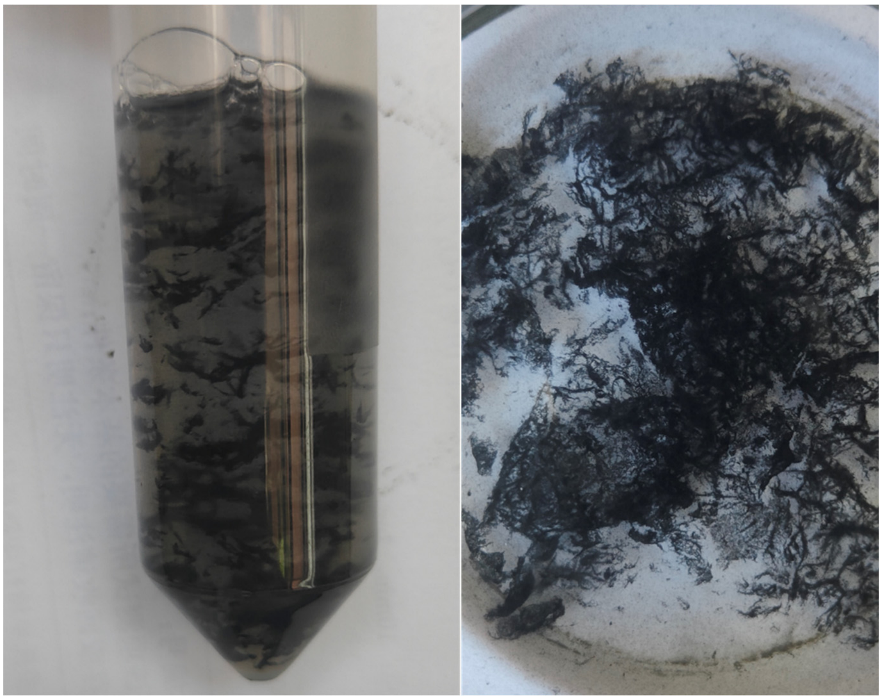

Figure S3. BC@S in the system after nine sterilization cycles.

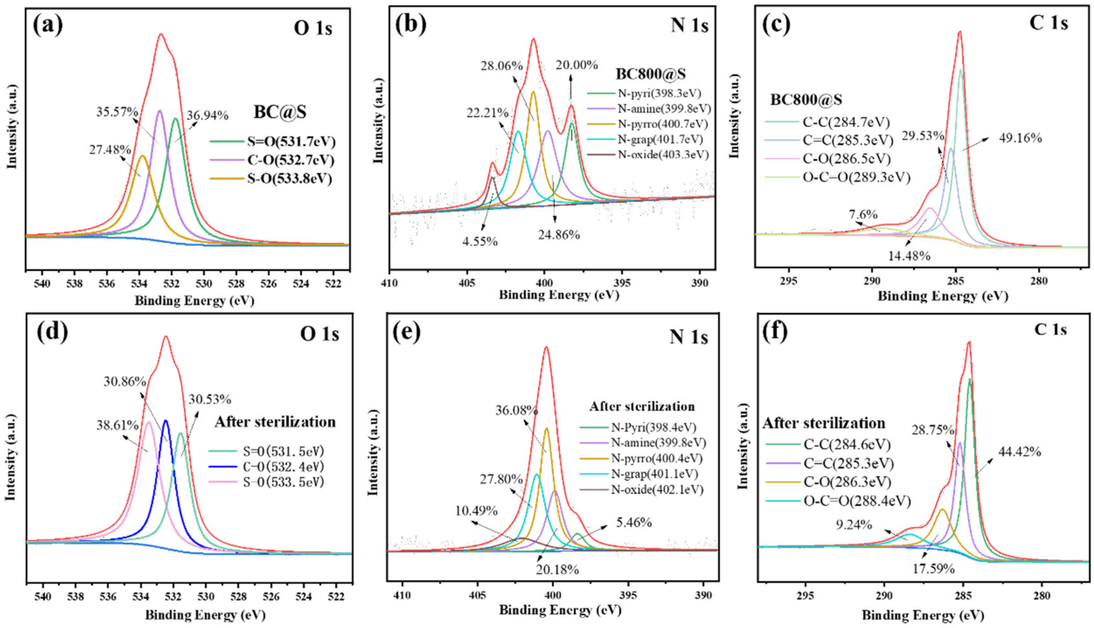

Figure S4. XPS spectrum of C 1s, O 1s and N 1s spectrum of BC@S (**a**–**c**) and After sterilization (**d**–**f**).
